# Theory of Mind Profiles in Children With Autism Spectrum Disorder: Adaptive/Social Skills and Pragmatic Competence

**DOI:** 10.3389/fpsyg.2020.567401

**Published:** 2020-09-17

**Authors:** Belen Rosello, Carmen Berenguer, Inmaculada Baixauli, Rosa García, Ana Miranda

**Affiliations:** ^1^Developmental and Educational Psychology, University of Valencia, Valencia, Spain; ^2^Occupational Sciences, Speech Language Therapy, Developmental and Educational Psychology Department, Catholic University of Valencia, Valencia, Spain; ^3^Developmental, Social, Educational Psychology and Methodology, Universitat Jaume I, Castellón de la Plana, Spain

**Keywords:** autism, adaptative skills, theory of mind, social, pragmatic competence

## Abstract

Theory of Mind (ToM) is one of the most relevant concepts in the field of social cognition, particularly in the case of Autism Spectrum Disorders (ASD). Literature showing that individuals with ASD display deficits in ToM is extensive and robust. However, some related issues deserve more research: the heterogeneous profile of ToM abilities in children with ASD and the association between different levels of ToM development and social, pragmatic, and adaptive behaviors in everyday life. The first objective of this study was to identify profiles of children with ASD without intellectual disability (ID), based on explicit and applied ToM knowledge, and compare these profiles with a group of children with typical development (TD). A second objective was to determine differences in symptom severity, adaptive/social behavior, and pragmatic abilities between the profiles identified. Fifty-two children with a clinical diagnosis of ASD without ID and 37 children with TD performed neuropsychological ToM tasks and two vocabulary and memory tests. In addition, all of their mothers completed different questionnaires about applied ToM abilities, severity of ASD symptoms, adaptive/social skills, and pragmatic competence. Two subgroups were identified in the cluster analysis carried out with explicit and applied ToM indicators. The “Lower ToM abilities” profile obtained significantly lower scores than the “Higher ToM abilities” profile on all the ToM measures. Furthermore, the analysis of covariance, controlling for vocabulary and working memory (ANCOVAs), showed statistically significant differences in applied ToM abilities between the two groups of children with ASD without ID and the group with TD. However, only the group with “Higher ToM abilities” achieved similar performance to the TD group on the verbal task of explicit ToM knowledge. Finally, the “Lower ToM abilities” cluster obtained significantly higher scores on autism symptoms (social and communication domains) and lower scores on adaptive behavior and pragmatic skills than the cluster with “Higher ToM abilities.” Taken together, these findings have implications for understanding the heterogeneity in ToM skills in children with ASD without ID, and their differential impact on social, communicative, and adaptive behaviors.

## Introduction

The Theory of Mind (ToM) is a broad, complex, and multifaceted construct, defined as the ability to attribute mental states (beliefs, desires, intentions) to oneself and to others, making it possible to explain and predict behavior (Premack and Woodruff, 1978). For decades, authors have argued that ToM deficits are prevalent in Autism Spectrum Disorder (ASD) (Baron-Cohen et al., 1985), a neurodevelopmental condition characterized by persistent communication and social interaction difficulties, restricted interests, and the presence of repetitive behaviors (American Psychiatric Association, 2013). Robust empirical findings confirm these ToM impairments in ASD (Kimhi, 2014), based on inferior performance on assessment tasks.

Autism Spectrum Disorders has a very heterogeneous range of symptoms with varying degrees of severity. Similarly, performance on tests assessing ToM skills is not uniform either. A key factor influencing this variability has to do with the ToM component being assessed and the type of task used for this purpose. Research currently supports the subdivision of ToM into implicit and explicit components that describe different aspects of social stimulus processing (Frith and Frith, 2012). On the one hand, explicit ToM skills refer to a conceptual, logical, and controlled ToM knowledge, which is distinguished by sequential and conscious processing (Satpute and Liberman, 2006; Frith and Frith, 2012). Tasks with clear instructions such as classic first- and second-order false beliefs would be paradigmatic examples of procedures for evaluating this explicit component. On the other hand, the implicit component of the ToM acts quickly, spontaneously, and unconsciously. It allows the correct anticipation of behavior without a deliberate reflection on the mental state of the other. In this regard, the tasks involving the categorization of facial expressions according to the emotion expressed are methods for evaluating implicit competence.

### Explicit and Applied Theory of Mind in ASD

In general, research has found that people with ASD without intellectual disabilities (ID) tend to perform better on explicit ToM tasks (Happé, 1995; Senju, 2012, 2013). This has been demonstrated through the use of standard first- and second-order false belief tests (Dahlgren and Trillingsgaard, 1996; Baron-Cohen, 2001; Cantio et al., 2018), and even with complex, advanced-level tasks (e.g., Director Task), where adolescents with ASD have been found to perform on par with the typically developing (TD) group (Barendse et al., 2018). In contrast, performance was significantly lower on tasks of an implicit nature, such as those based on facial emotion perception and categorization without the aid of contextual cues (Harms et al., 2010; Uljarevic and Hamilton, 2013; Lozier et al., 2014; Schaller and Rauh, 2017), free verbal judgments about social situations (Callenmark et al., 2014), or gaze patterns, assessed by eye-tracking, which reflect spontaneous attributions of false beliefs (Zhou et al., 2019).

A question that has been widely discussed in research is whether, regardless of the type of task and the ToM component assessed, there is a clear discrepancy between the performance of people with ASD on ‘laboratory’ measurements and their application of ToM in natural everyday environments (Senju et al., 2009; Scheeren et al., 2013). Hutchins et al. (2016, p. 98) defined applied ToM as “the ability to deploy ToM knowledge to successfully address ToM dilemmas as they are presented in real-world samples of behavior.” It has been observed, for example, that people with ASD can succeed on false belief tasks, but they fail when they have to act spontaneously based on this knowledge, i.e., when they have to demonstrate applied ToM (Senju, 2012; Livingston and Happé, 2017).

There could be several reasons for this discrepancy in the results. Undoubtedly, real-life situations are more complex and dynamic in terms of information processing. As Hutchins et al. (2016, p. 98) highlighted, “applied ToM competence is ostensibly affected by a variety of endogenous (e.g., executive functioning, motivation, and sensitivity) and exogenous (e.g., physical setting) factors.” Clearly, during everyday social interactions, people with ASD are exposed to a continuous stream of ToM challenges with varying demands. The social cues are more unpredictable and ambiguous, and they take place under time pressure with limited information and cognitive resources. The large number of verbal and non-verbal contextual cues make them difficult to process automatically, causing congestion that acts as a bottleneck in the processing of social stimuli. This problem is compounded by social patterns that have not been adequately developed (Schaller and Rauh, 2017). These difficulties with applied ToM are consistent with findings showing that training in the attribution of mental states in formal situations does not necessarily guarantee better social adaptation of people with ASD (Begeer et al., 2011; Senju, 2013). For this reason, procedures for the assessment of mental skills have been designed with greater ecological validity, attempting to capture the application of ToM to the real world in everyday life. Questionnaires such as the “Theory of Mind Inventory” (ToMI) (Hutchins et al., 2011) have made it possible to identify disorders in children with ASD with larger effect sizes than those obtained from the administration of explicit proficiency tests (Berenguer et al., 2018).

Another reason for the inconsistent findings may be the use of measures that have been designed to assess a broad spectrum of ToM skills, ranging from understanding basic mental states to skills at a more advanced level of development (Steele et al., 2003). The controlled condition where the assessment is conducted would also influence the results. Thus, the use of simple structured tasks with explicit instructions and limited options decreases the social cognition demands, which would favor successful results. Other possible sources of variability in ToM results in ASD would be cognitive ability, given that ToM is a meta-representational skill dependent on general domain cognitive skills (Pellicano, 2010; Pruett et al., 2015), or even other deficient processes in ASD, such as executive functions (Miranda et al., 2017; Demetriou et al., 2018). It has also been documented that ToM task performance is closely related to language skills, particularly receptive vocabulary and complementation syntax (Tager-Flusberg, 2001). Language proficiency would act as a compensatory mechanism to facilitate task achievement, but it would not imply the mastery of genuine and mature ToM.

### ToM and Adaptive/Social Skills

In general, ToM skills, the ability to share feelings, exchange ideas, and anticipate others’ behavior, are essential for social life (Zhou et al., 2019). Successful social functioning requires an understanding of other people’s emotions, intentions, beliefs, and knowledge. However, although deficits in mind-reading skills may reasonably explain, at least in part, the social difficulties experienced by people with ASD, research findings are inconsistent.

Pioneering studies such as the one by Fombonne et al. (1994) sought precisely to describe the associations between adaptive social skills, assessed by parental reports on a subset of items from the Vineland Adaptive Behavior Scales (Sparrow et al., 1984), and performance on false belief tasks. Specifically, in the study by Fombonne et al. (1994), participants who succeeded in overcoming social cognition tasks were older, showed higher intellectual ability, and performed better on social and adaptive behaviors involving understanding minds. However, when their verbal ability was taken into account, these specific differences were no longer significant.

Later, other studies using measures of social understanding (false belief understanding, affective perspective-taking) and measures of social responsiveness and social interaction (level of engagement with peers on the playground and prosocial behavior in a structured laboratory task) found that, in children with autism, initiating joint attention and empathy were strongly related to both measures of social interaction competence (Travis et al., 2001). Similar results were obtained when using teachers’ ratings of peer interaction skills, which showed a significant correlation with the scores obtained on false belief tasks by children with ASD (Peterson et al., 2007).

To the same end, Tager-Flusberg (2001) applied a battery of various ToM tasks (including symbolic play, moral judgment, and false belief) to a large sample of participants with ASD, and they found a significant association between ToM skills and social competence, again assessed with the Vineland social scale (Sparrow et al., 1984). More recently, Bishop-Fitzpatrick et al. (2017), in a cross-sectional study, showed that better performance on second-order false belief tasks was associated with better socio-adaptive behavior and fewer social problems. Mazza et al. (2017), using mediation analysis, warned that ToM plays a key role in the development of social skills, and that the lack of ToM competence in children with autism alters their competent social behavior. Thus, they concluded that the ability to understand emotions and beliefs is necessary in order to display appropriate social behavior. Finally, Altschuler et al. (2018) reported a positive relationship between affective ToM (ability to infer other people’s emotions) and social symptoms characteristic of ASD. In other words, affective ToM predicted the severity of social symptoms, but not social functioning in a broad sense. In the same study, no type or level of ToM (basic or advanced) was able to predict the social behavior described by the parents.

Not all research has identified this positive association between ToM and social competence. For example, Prior et al. (1990) could not find a relationship between performance on false belief tasks and caregivers’ estimates of the social skills of their children with autism. Similarly, although in Joseph and Tager-Flusberg’s (2004) study, ToM and executive functions could explain significant variance on the Communication section of the ADOS-G (Lord et al., 2000), this effect did not occur in the Social Interaction section. That is, the executive functions and the ToM were more strongly associated with communicative functioning than with social functioning. Moreover, it has been possible to identify a subgroup or profile of individuals with ASD who, in spite of manifesting continuous difficulties in understanding the mind of the other, exhibited few social affectation symptoms (Livingston et al., 2019). This subgroup, called “high compensators,” presented characteristics such as a higher verbal intelligence quotient (IQ) and better executive functioning skills, among other features.

In addition, in longitudinal studies like the one by Bennett et al. (2013), although language, non-verbal IQ, and ToM predicted a relatively small but significant amount of variance in adaptive functioning, ToM was not uniquely predictive of variance in adaptive socialization in early adolescence after controlling for IQ. Nor was this predictive power of ToM found in the study by Peterson et al. (2016), who noticed that neither language ability nor ToM directly predicted peer social skills.

The inconsistency in the results seems to indicate that ToM is necessary but not sufficient to explain social competence. Therefore, an attempt has been made to identify other factors that, along with ToM skills, can better justify social functioning deficits in ASD. Thus, studies have shown that ToM competence combined with pragmatic language skills can predict and directly and indirectly influence the socialization of children with ASD without intellectual disabilities (Berenguer et al., 2018). These relationships are to be expected, given the profile of vulnerability that children with ASD present in the pragmatic area, a universal deficit in the disorder (Lam, 2014).

### ToM and Pragmatic Ability in ASD

Theory of Mind and the pragmatic dimension of language are closely intertwined, and several findings from different approaches support this relationship. From a developmental perspective, it has been raised that ToM and pragmatics are co-evolved functions (Westra and Carruthers, 2017). From a psycholinguistic framework, no account can be given of key pragmatic notions like indirect speech acts, deictic expressions, presuppositions, pronoun reference or irony, in the absence of the involvement of ToM (Cummings, 2013). Finally, from a neurobiological point of view, a significant overlap has been found between the neural basis of ToM and that of narrative comprehension (Mar, 2011), which is directly related to pragmatic skill (Botting, 2002). All these arguments have led to conclude that “ToM and pragmatic aspects of language are so fused that they cannot be separable” (Kobayashi, 2018, p. 118). In this regard, O’Neill (2012) established a pragmatic taxonomy in which “mindful pragmatics” was considered, that is, the uses of language that require adopting the perspective of the listener, such as engaging in a conversation or elaborating a speech. In both situations, the information needs of the receiver must be monitored and adapted to his/her perspective. Ultimately, the correct interpretation of the intentions and beliefs of the interlocutor in relation to the context is absolutely essential for good development of pragmatic communication.

Although still scarce, most studies on the subject demonstrate a significant association between mind-reading skills and pragmatic competence. Thus, correlations have been found in children with autism – but not in children with developmental delay – between performance on ToM tasks and the ability to respond to a conversational partner with new, relevant, and contingent information (Capps et al., 1998). The same significant association has been found between understanding of first-order false belief tasks and various narrative properties, such as the use of evaluative statements (Capps et al., 2000) and referential cohesion (Kuijper et al., 2015). Specifically, in relation to discourse, longitudinal studies have found that ToM contributed unique variance in discourse skills beyond the contribution of language competence (Hale and Tager-Flusberg, 2005). Furthermore, a mediating role of ToM has been identified in the association between language ability at the age of 6–8 years and adaptive communication measured 6 years later, which suggests that “structural language (grammar and vocabulary), ToM and later adaptive communication are related over the course of development in children with ASD” (Bennett et al., 2013, p. 17).

### Symptom Severity and ToM

Research has shown an association between greater ToM deficits and ASD symptom severity in terms of social communication difficulties and restricted and repetitive behaviors. A study by Shimoni et al. (2012) found that clinical assessment of autistic symptoms in children with Asperger Syndrome/High Functioning Autism was negatively correlated with ToM measures, obtained through The Social Attribution task (SAT) (Klin, 2000). Statistically significant correlations were found for the Pertinence and Salience indices, and for measures of the ADI-R (Rutter et al., 2006). In other words, more autistic symptoms were related to more non-pertinent propositions and fewer social elements identified.

Subsequently, Hoogenhout and Malcolm-Smith (2017), using hierarchical cluster analysis, determined that ToM skills were capable of reliably discriminating ASD severity levels, and the three clusters they identified (severe, moderate, and mild ASD) were strongly associated with the level of support required, as indicated by the type of school environment. These results agree with those reported by Aljunied and Frederikson (2011), who found that a ToM index, combined with IQ measures, contributed significantly to the categorization of children with ASD in three types of educational support. Particularly, for children with less severe needs, those who did not need any additional support were differentiated from those who did by ToM measures.

Finally, using structural equation modeling, and accounting for ToM and executive functions (EF) in one model, Jones et al. (2018) established that mind-reading difficulties were associated with more severe social communication symptoms and restrictive and repetitive behaviors, in adolescents with ASD. It is noteworthy that the strength of associations between social communication and ToM and between restrictive and repetitive behaviors and ToM were similar. This last finding contrasts with the results of other studies that did not find any significant correlation between ToM and restrictive and repetitive behaviors (Joseph and Tager-Flusberg, 2004; Cantio et al., 2016). These inconsistencies are explained according to the procedure used to evaluate the behavior symptomatology, mainly through observation or parent-interview. In contrast, Jones et al. (2018) used a targeted questionnaire designed to gather information about the breath of restricted and repetitive behaviors observed in ASD. It is concluded that “a bewildering social world due to impoverished mentalizing abilities could lead to that kind of behaviors that lessen anxiety and reduce confusion” (Jones et al., 2018, p. 103). Consequently, impairments in understanding the social world could promote the emergence of idiosyncratic and unusually intense interests and repetitive behaviors.

In summary, ToM is a complex construct that has not been used consistently in research, which has led to considerable variability in the evaluation tasks and mixed literature results. It is likely that the divergent results at least partly depend on the assessment demands and the cognitive level of the individuals being assessed. Therefore, measures of explicit ToM competence and applied ToM competence, along with different levels of ToM skills and cognitive levels, should be taken into account to identify more homogeneous profiles. A cluster analysis may be an appropriate methodology to establish different profiles of mind-reading skills within ASD when attempting to analyze the relationships between ToM and other common difficulties in this disorder, such as pragmatic difficulties or social adjustment problems.

Consequently, the first objective of this study was to identify profiles of children with ASD without intellectual disability (ID), based on explicit and applied ToM knowledge, comparing these profiles with a group of children with typical development (TD). We hypothesize that the profiles identified in children with ASD will differ on ToM skills of different types (explicit and applied), and that these children, even the best performing profile, will have a lower level of development than the TD group. A second objective was to examine differences in ASD symptom severity, social and adaptive behavior, and pragmatic abilities among the identified profiles. We expect that, based on the central role of ToM deficits in ASD, the profile with lower ToM abilities will show more severe symptoms and lower socio-adaptive and pragmatic skills.

## Materials and Methods

### Participants

This study included 52 children with ASD without intellectual disability (ID) and 37 children with typical development (TD). The two groups of children were between 7 and 11 years old, and they had an intellectual functioning within the limits of normality on the K-BIT (Kaufman and Kaufman, 2000).

The group of children with ASD had received a clinical diagnosis of an autism spectrum condition in hospitals and medical centers by Psychiatry and Child Neurology services in the Valencian community at ages ranging between 2 years and 11 months and 6 years old. According to the protocol for the ASD diagnosis, the Diagnostic and Statistical Manual of Mental Disorders criteria for ASD from the fourth edition (DSM-IV; American Psychiatric Association, 1994), the Autistic Diagnostic Interview—Revised (ADI-R; Rutter et al., 2006), and/or the Autism Diagnostic Observation Schedule-WPS (ADOS-WPS; Lord et al., 1999) were administered by a multidisciplinary team. In order to confirm the ASD diagnosis for the present study, the Social Communication Questionnaire (SCQ; Rutter et al., 2003) and the Autism Diagnostic Interview-revised (ADI-R; Rutter et al., 2006) were administered, taking into account the recommended cut-off points. These two instruments were administered to the parents by a clinical psychologist from the research team who had been accredited in their application. Likewise, all the children met the strict diagnostic criteria for ASD from the fifth edition of the Diagnostic and Statistical Manual of Mental Disorders (DSM-5; American Psychiatric Association, 2013), based on information reported by teachers and parents. Both informants, through interviews with a clinical psychologist, rated the severity of the criteria in the two ASD dimensions on scales ranging from 0 to 3 points (0 represents “almost never,” 1 “sometimes,” 2 “often,” and 3 “many times”).

Regarding the school modality, three children with ASD (5.8%) were attending school in regular classrooms full time without educational support; 29 children (55.7%) attended regular classrooms but received educational support for their specific needs in the school; and finally, 20 children (38.5%) were placed in the Communication and Language classroom modality. In other words, according to the DSM-5 (American Psychiatric Association, 2013), the support required by the participants corresponded to level 1 severity. Furthermore, 32.7% of the children with ASD were taking antipsychotic medication (mostly risperidone) for behavioral problems and irritability symptoms.

The typically developing children were in the same schools as the clinical sample in the study. They had no history of psychopathology or referral to pediatric mental health units (USMI), according to the information found in the school records, and they did not meet DSM-5 criteria for ASD on the screening carried out before beginning the evaluation. None of them was taking any psychoactive medication.

The exclusion criteria for the children who participated in this study were evaluated through an extensive anamnesis carried out with the families. They included neurological or genetic diseases, brain lesions, sensory, auditory, or motor deficits, and an IQ below 80.

Both groups of children, with ASD and with TD, were matched on age [*t*(89) = −0.15, *p* = 0.88], IQ [*t*(89) = −0.28, *p* = 0.78], and their level on a Vocabulary subtest from the WISC-IV (Wechsler, 2003) [*t*(89) = −1.04, *p* = 0.30].

### Measures

The selection of the measures used was primarily based on the following criteria: utility and relevance according to the objectives of this study, translation and adaptation to Spanish and good psychometric properties.

#### Explicit and Applied ToM Knowledge

The subtests of Affect Recognition and Theory of Mind, which are included in the Social Perception domain of the NEPSY–II (A Developmental Neuropsychological Assessment Battery, Korkman et al., 2007), were administered to all the children to assess their explicit ToM knowledge. The first subtest, Affect Recognition (AR), aims to evaluate the ability to identify emotions (happy, sad, anger, fear, disgust, and neutral emotion) through photographs of children’s faces. The second subtest, Theory of Mind, contains two parts. The first part (verbal task) includes 15 items that assess the subject’s ability to understand beliefs, intentions, thoughts, and feelings that are different from their own. The child is read various scenarios or shown pictures, and s/he is then asked to correctly answer questions that require knowledge about another individual’s point of view. The second part (contextual task) includes 5 items that assess the subject’s ability to put him/herself in the place of one of the characters and think about what s/he is feeling in a situation represented in a drawing. The child is shown a picture depicting a social context and asked to select one photograph from four options that depicts the appropriate affect of one of the people in the picture. Higher scores on both Nepsy-II tests indicate greater development of theory of mind skills. Many studies have reported reliability data for all the scales, and there is also evidence of convergent and discriminant validity of the NEPSY battery (Korkman et al., 2007).

To evaluate the application of ToM skills, the parents completed the Theory of Mind Inventory (ToMI; Hutchins et al., 2014; Spanish adaptation by Pujals et al., 2016). It comprises 42 items distributed in three scales, and each item is scored from 0 to 20 points, with 5 response alternatives ranging from “definitely not” to “definitely.” The early subscale assesses skills for understanding basic emotions. The basic subscale includes understanding mental terms and the distinction between physical and mental representations. Finally, the advanced subscale, which was used in this study, assesses second-order beliefs (i.e., “My child understands that people can be wrong about what other people want”) and competence in understanding inferences and complex social judgments (i.e., “My child understands the difference between a friend teasing in a nice way and a bully making fun of someone in a mean way”). High scores indicate good perception in the development of theory of mind skills. The ToMI has adequate validity, good internal consistency, and test-retest reliability. It has also shown excellent sensitivity (0.90) and specificity (0.90) (Hutchins et al., 2011; Pujals et al., 2016).

#### Psychological and Behavioral Adjustment

The SDQ questionnaire (SDQ-Cas-Goodman, 1997; adapted to Spanish by Rodríguez-Hernández et al., 2012) was filled out by the parents to assess a broad range of mental health symptoms. It contains a total of 25 items grouped in five subscales (emotional symptoms, behavioral problems, hyperactivity/attention problems, peer relationship problems, and prosocial behavior problems). Specifically, four of the five subscales are scored in a similar way, with higher scores indicating a greater likelihood of significant problems, whereas the prosocial subscale provides a reverse score where higher scores indicate more prosocial behaviors or strengths. In this study, we used the subscale of peer relationship problems, which contains 5 items (i.e., “Rather solitary, tends to play alone), and the subscale of prosocial behavior, which also has 5 items (i.e., “Helpful if someone is hurt, upset, or feeling ill”).

The SDQ has shown good statistical and psychometric properties, with Cronbach’s alpha values above 0.70 (Goodman, 2001), confirmed in the Spanish population (0.76) (Rodríguez-Hernández et al., 2012). It also obtained acceptable to high internal consistency in the current study (Cronbach’s α = 0.74–0.80 between subscales).

#### Adaptive/Social Skills

The Vineland Adaptive Behavior Scale (VABS-II ed; Sparrow et al., 2005) was filled out by parents to evaluate the adaptive capacity of their children. It includes four fundamental domains: communication, daily living skills, socialization, and motor skills. It has another domain that extracts an index of maladaptive behavior. For this study, the scores in two domains were used, daily living skills and socialization skills. Daily living skills describe personal (e.g., eating, dressing, and hygiene), domestic (e.g., household tasks performed), and community (e.g., using money, answering the phone) tasks, and the socialization scale also includes three subscales that describe interpersonal relationships, play and leisure time, and coping skills.

The Vineland-II scale has been widely used in people with ASD to evaluate social maturity. It has solid psychometric properties, with high test-retest reliability (α = 0.98) (Sparrow et al., 2005).

#### Pragmatic Abilities/Pragmatic Competence

The Children’s Communication Checklist (CCC-2; Bishop, 2003) provides information about communication characteristics in subjects from 4 to 11 years old. The frequency of the behaviors described in each item included in the CCC-2 is rated on a 4-point scale; a high score indicates greater communication problems. In addition, the 70 items included in the CCC-2 are grouped in 10 subscales that measure different communicative aspects. The first block assesses the structural aspects of language and has four subscales (*speech*, *syntax*, *semantics*, and *coherence*). The second block evaluates the pragmatic aspects of language and also has four subscales (*inappropriate initiation*, *stereotyped language*, *use of context*, and *non-verbal communication*). Finally, the last block contains two subscales designed to evaluate the typical features of ASD (*social relationships and interests*). In the present study, we used the pragmatic composite index (PCI), which is obtained by adding together the scores on the coherence, inappropriate initiation, stereotyped language, use of context, and non-verbal communication subscales. This specific grouping, although not contemplated in the CCC-2, has been used in other previous studies (Helland, 2014). The CCC-2, which in this study was filled out by the parents, presents good internal consistency that ranges between 0.66 and 0.80 (Bishop, 2003).

#### Severity of ASD Symptoms

The severity of ASD symptoms was assessed with the Social Communication Questionnaire (SCQ; Rutter et al., 2003), which is based on a semi-structured parent interview used for the diagnostic evaluation of children with suspected ASD. It provides information about three domains of autistic symptoms: reciprocal social interaction (i.e., “Does your son/daughter have specific friends or a close friend?”); communication (i.e., “Can you have a conversation with him/her that flows both ways and requires taking turns speaking or elaborating on what was said before?”); and restricted/repetitive behaviors (i.e., “Has s/he ever shown more interest in the parts of a toy or object [for example, turning the wheels on a car] than in using the toy itself?”). The SCQ has good psychometric properties (Cronbach’s alpha of 0.84–0.93 across age groups and a Cronbach’s alpha of 0.81–0.92 across diagnostic groups) (Rutter et al., 2003). In this study, the Cronbach’s alpha for the questionnaire was 0.78, which is similar to what Rutter et al. (2003) reported.

### Procedure

This research was performed in accordance with the ethical standards of the Research Ethics Committee of the University of Valencia, which is regulated by Ethical Principles for Medical Research Involving Human Subjects (Declaration of Helsinki 1964, World Medical Association, 2013). Likewise, it received authorization from the Board of Education of the Valencian Government to access the schools and locate the participants.

The evaluation was carried out in the schools where the children were enrolled, in specially prepared spaces that met optimal conditions for psychoeducational assessment. The informed oral and written consent of the parents of all the participants was also obtained after informing them about the research proposal. The children were evaluated during school hours, without interfering with the basic curricular activities. The intelligence test and the two tests from the social perception domain were administered to all the children individually by trained examiners. The parents (mostly mothers) provided information about their children’s ToM skills in daily life contexts, ASD symptoms, and adaptive/social skills. The teachers-tutors filled out the questionnaire selected to assess EF.

### Data Analyses

The statistical analyses were performed with the statistical program for the Social Sciences [SPSS v 24.0 (SPSS)]. Preliminary analyses checked all data for multicollinearity and multivariate outliers. The asymmetry and kurtosis data indicate that most of the variables followed a normal distribution (all values between −1 and 1). Variables that did not show a normal distribution were transformed using square-root transformation.

To examine distinct profiles (i.e., subgroups) of Theory of Mind abilities in ASD, we performed hierarchical cluster analysis. The input for this analysis included three variables from the social perception domain of the NEPSY-II battery: Emotion recognition, Verbal task of ToM, and Contextual task of ToM; and three variables from the Theory of Mind Inventory (ToMI): Early scale, Basic scale, and Advanced scale. Moreover, the variables were standardized to *z*-scores.

We evaluated hierarchical clustering using multiple internal validity measures. Specifically, we varied the number of clusters from two to three, and the optimal N-cluster solution was determined on the basis of visual inspection of the dendrogram figure and the agglomeration coefficients.

Additionally, we also carried out the same procedure with non-hierarchical clustering, namely, *K*-means, because this procedure allows us to specify the number of clusters in advance. Lastly, in order to fit the optimal cluster analysis solution, we used the variance ratio criterion (VRC) for each selected cluster. The VRC refers to the ratio of the ‘within variance’ (variance explained by the typology) and ‘between variance,’ corrected for the number of clusters and responses. The two-cluster solution seemed to be optimal in the hierarchical cluster analysis, based on Ward’s method, and the VRC showed a lower score for two solutions (Cohen-Addad et al., 2019).

After analyzing the resulting dendrogram and data, the decision was made to group the children in two clusters, controlling for vocabulary and working memory (ANCOVAs). We labeled each of the ASD subgroups based on the patterns of functioning across domains of ToM abilities. Then we checked the possible differences between the Clusters obtained and a control group with TD.

Finally, one-way analyses of variance (ANOVAs) were conducted to determine the differences between the children in the cluster groups on the following measures: the Strengths and Difficulties Questionnaire- SDQ (Peers problems and Prosocial scale); the Vineland Adaptive Behavior Scales- VABS-II (Daily life skills and Socialization domains); the Children’s Communication Checklist -CCC-2 (Pragmatic Index); and the Social Communication Questionnaire-SCQ (Social, Communication, and Stereotyped behavior scales). For the ANOVAs, the level of significance was set at *p* < 0.004, after applying the Bonferroni correction. The proportion of total variance accounted for by the independent variables was calculated using partial eta squared [according to Cohen (1988): eta squared, 0.06 = small; 0.06–0.14 = medium, 0.14 = large].

## Results

### Profiles of Children With ASD Without Intellectual Disability (ID) Comparison of Profiles With Children With Typical Development (TD)

The first goal of the analysis was to examine whether children with ASD were more likely to cluster into a single group or multiple groups on the basis of measures of ToM skills.

Results from the hierarchical cluster analysis with the children’s ToM abilities determined an optimal number of clusters in two groupings, distinguished by the tendency of their scores on the variables included in the analysis: TOM explicit knowledge (emotion recognition, verbal, and contextual ToM) and applied knowledge (Early, Basic, and Advanced ToMI). Cluster 1 (*n* = 22; 42.30%) presented higher scores on all the variables of theory of mind skills, on both applied ToM abilities and explicit ToM abilities. By contrast, Cluster 2 (*n* = 30; 57.69%) showed lower scores than Cluster 1 on all the ToM skills measured.

Analyses of covariance, controlling for vocabulary and working memory (ANCOVAs), were then conducted to determine the significant differences between the two clusters in the theory of mind skills considered. After applying Bonferroni correction, children classified in Cluster 1 obtained scores that were statistically different from Cluster 2 on most of the measured variables, with the comparisons showing moderate to large effect sizes on Verbal ToM and Early, Basic, and Advanced ToMI: Verbal-ToM, *F*_1,50_ = 50.39, *p* < 0.001, $\eta_{\text{p}}^{2}$ = 0.50; 001; Early-ToMI, *F*_1,50_ = 19.25, *p* < 0.001, $\eta_{\text{p}}^{2}$ = 0.28; Basic-ToMI, *F*_1,50_ = 44.11, *p* < 0.001, $\eta_{\text{p}}^{2}$ = 0.47; Advanced-ToMI, *F*_1,50_ = 9.99, *p* < 0.001, $\eta_{\text{p}}^{2}$ = 0.16. The differences between the two groups did not reach statistical significance after applying the Bonferroni correction on the effect sizes for Emotion Recognition ($\eta_{\text{p}}^{2}$ = 0.09) and the contextual ToM task ($\eta_{\text{p}}^{2}$ = 0.11).

**FIGURE 1:**
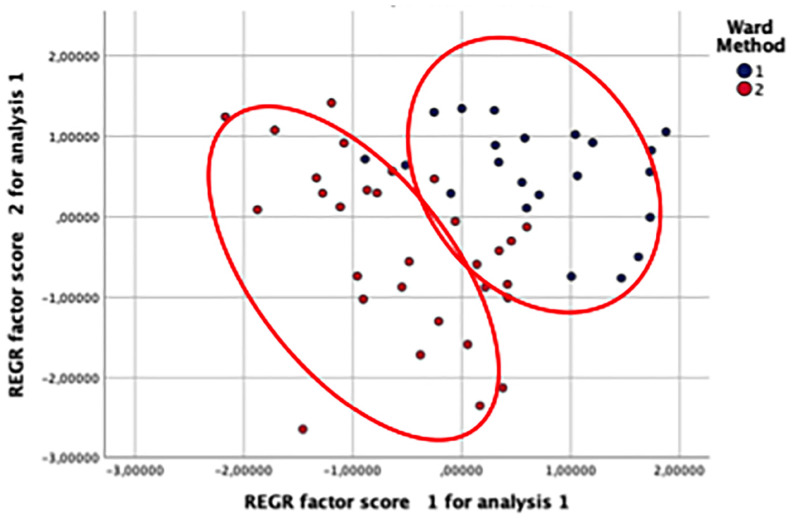
Scatterplot of pairwise comparisons between clusters on each TOM subscale.

Based on the described patterns of functioning across domains of ToM abilities, Cluster 1 was labeled “Higher ToM skills,” and Cluster 2 was called the “Lower ToM skills” group (see Table 1 and Figure 1).

**TABLE 1:** Means, standard deviations (SD) of TOM skills for the two clusters obtained, and statistically significant differences between the two clusters (Higher TOM skills and Lower TOM skills).

Measure	Cluster 1 Higher TOM skills (*n* = 22) *M (SD)*	Cluster 2 Lower TOM skills (*n* = 30) *M (SD)*	*F*_(1,50)_	*p*	$\eta_{\text{p}}^{2}$
Emotion Re	25.18 (3.14)	22.70 (4.43)	5.02	0.029	0.09
Verbal TOM	17.00 (2.74)	11.83 (2.47)	50.39	0.000*	0.50
Contextual TOM	4.54 (0.80)	3.51 (1.85)	5.95	0.018	0.11
Early ToMI	16.58 (3.19)	13.00 (2.67)	19.25	0.000*	0.28
Basic ToMI	15.57 (2.24)	10.98 (2.60)	44.11	0.000*	0.47
Advanced ToMI	9.43 (2.84)	6.99 (2.73)	9.79	0.003*	0.16

*Emotion Re, emotion recognition; Verbal TOM, theory of mind-Verbal; Contextual TOM, theory of mind- contextual; ToMI, theory of mind inventory*. *p < 0.008 (Bonferroni correction).

Additionally, the results of the Bartlett sphericity test indicate that the variables were sufficiently intercorrelated [χ^2^(15) = 78.25; *p* < 0.001], which is an important requirement for subsequent multivariate analysis.

Multivariate Analysis of Variance (MANOVA) was conducted to analyze differences between Cluster 1 “Higher ToM abilities,” Cluster 2 “Lower ToM abilities,” and the TD group on the Emotion recognition and social perception subscales of the NEPSY-II (explicit ToM knowledge) and the ToMI inventory scales (applied knowledge). The MANOVA conducted to assess the main group effect among the three groups was statistically significant [Wilk‘s Lambda (Λ) = 0.07, *F*_(12,162)_ = 37.23, *p* < 0.001, $\eta_{\text{p}}^{2}$ = 0.73]. ANOVAs showed significant differences on the NEPSY subscales: Emotion recognition, *F*_2,86_ = 22.46, *p* < 0.001, $\eta_{\text{p}}^{2}$ = 0.34; Verbal-ToM, *F*_2,86_ = 52.81, *p* < 0.001, $\eta_{\text{p}}^{2}$ = 0.55; Contextual-ToM, *F*_2,86_ = 9.43, *p* < 0.001, $\eta_{\text{p}}^{2}$ = 0.18. Statistically significant differences were also found on the applied ToM tasks (ToMI): Early-ToMI, *F*_2,86_ = 54.36, *p* < 0.001, $\eta_{\text{p}}^{2}$ = 0.55; Basic-ToMI, *F*_2,86_ = 132.51, *p* < 0.001, $\eta_{\text{p}}^{2}$ = 0.75; Advanced-ToMI, *F*_2,86_ = 139.89, *p* < 0.001, $\eta_{\text{p}}^{2}$ = 0.76.

Bonferroni *post hoc* analyses showed statistically significant differences on the Verbal TOM task between Cluster 2 “Lower ToM abilities” and both Cluster 1 “Higher ToM abilities” and the TD group, whereas there were no significant differences between Cluster 1 “Higher ToM abilities” and the TD group. A similar pattern was observed on the Contextual ToM task, where there were no significant differences between Cluster 1 (“Higher ToM abilities”) and the TD group, but there were statistically significant differences between Cluster 2 “Lower ToM abilities” and both Cluster 1 “Higher ToM abilities” and the TD group. Finally, there were statistically significant differences between the TD group and both Cluster 1 and Cluster 2 on Early-ToMI, Basic-ToMI, and Advanced-ToMI (*p* < 0.001), with significant differences between the two Clusters of ASD children (“Higher” and “Lower ToM abilities”). Consequently, Cluster 1, in the comparison with the TD group, showed a profile of generalized deficits affecting both explicit and applied ToM skills. In contrast, the deficit of Cluster 2, in comparison with the TD group, was found in the application of ToM skills.

**FIGURE 2:**
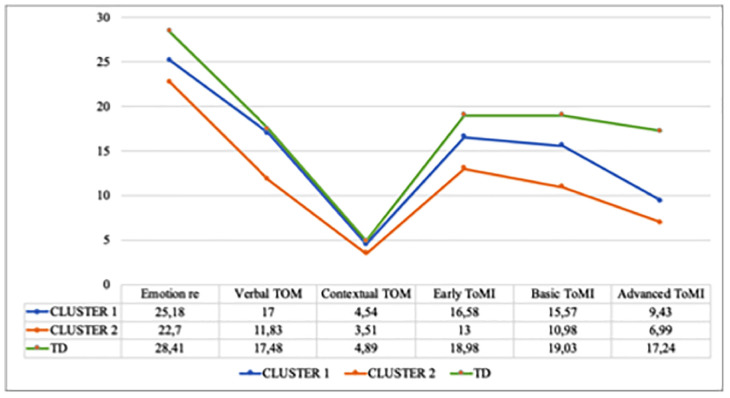
Means of Clusters 1, 2, and the typically developing group (TD) on the TOM children’s variables.

Figure 2 shows the comparisons of the mean scores of the two ASD clusters and the TD group.

### Differences in Severity of Symptoms, Social-Adaptive Behavior, and Pragmatic Abilities Across the Profiles of Children With ASD

Table 2 presents the comparison of two Clusters of ASD, ‘Lower ToM abilities” and ‘Higher ToM abilities,” on social-adaptive behavior, pragmatic abilities, and severity of symptoms. The analysis of variance revealed statistically significant differences between the two clusters in Daily life skills (VABS) *F*_1,50_ = 11.07, *p* = 0.002, $\eta_{\text{p}}^{2}$ = 0.18; Social domain (VABS) *F*_1,50_ = 15.27, *p* < 0.001, $\eta_{\text{p}}^{2}$ = 0.23; Pragmatic index (CCC) *F*_1,50_ = 16.48, *p* < 0.001, $\eta_{\text{p}}^{2}$ = 0.25; the Social symptoms domain (SCQ) *F*_1,50_ = 9.97, *p* = 0.003, $\eta_{\text{p}}^{2}$ = 0.17; and the Communication symptoms domain (SCQ) *F*_1,50_ = 14.61, *p* < 0.001, $\eta_{\text{p}}^{2}$ = 0.323. After applying the Bonferroni correction (*p* < 0.004), the variables that remained significant were the same: Daily life skills (VABS), Socialization skills (VABS), Pragmatic index (CCC), Social symptoms domain (SCQ), and Communication symptoms domain (SCQ).

**TABLE 2:** Means, standard deviations (SD) of social, adaptive behavior, and autism severity for the two clusters obtained (Higher TOM skills and Lower TOM skills), and statistically significant differences between them.

	Cluster 1 Higher TOM skills (*n* = 22)		Cluster 2 Lower TOM skills (*n* = 30)				
		
Measures	*M*	*SD*	*M*	*SD*	*F*_(1,50)_	*p*	$\eta_{\text{p}}^{2}$
SDQ_Peers	5.77	2.09	5.77	2.19	0.00	0.992	0.00
SDQ_Prosocial	6.46	2.69	5.23	1.94	3.63	0.061	0.07
VABS_Daily L	82.77	9.18	74.37	8.86	11.07	0.002*	0.18
VABS_Social	81.00	10.0	71.43	7.58	15.27	0.000*	0.23
CCC_Pragmatic I	23.77	8.62	15.20	6.61	16.48	0.000*	0.25
SCQ_Social	7.86	3.31	11.03	3.77	9.97	0.003*	0.17
SCQ_Communica	6.20	2.35	8.47	1.93	14.61	0.000*	0.23
SCQ_Stereotyp	4.65	2.06	4.93	1.93	0.25	0.617	0.01

**SDQ Peers, strengths and difficulties questionnaire peers problems scale; VABS_Daily L, vineland adaptive behavior scales_daily life skills; CCC_Pragmatic I, children’s communication checklist_pragmatic index; SCQ_Communica, social communication questionnaire_communication; SCQ_Stereotyp, stereotyped behavior scale. ^∗^p < 0.004 (Bonferroni correction).**

## Discussion

A critical target in ASD research is to identify homogenous subgroups to better understand neurodevelopmental patterns and design meaningful intervention strategies. In the past decade, several studies have used the methodological resource of cluster analysis to empirically derive ASD subtypes that share common cognitive and behavioral characteristics (Baeza-Velasco et al., 2014; Campbell et al., 2014; Hoogenhout and Malcolm-Smith, 2017). Following this approach, the first aim of the present study was to identify profiles of children with ASD without ID, based on measures of explicit and applied ToM knowledge. The cluster analysis carried out using different ToM measures made it possible to identify two profiles of children with ASD. One group, made up of 42.30% of the participants, showed better performance than the other group on all the variables of ToM abilities, and so it was labeled the “Higher ToM abilities” group. The other group had the lowest ToM performance and consisted of 57.69% of the children with ASD, and so it was called the “Lower ToM abilities” group. Moreover, both clusters differed significantly on the explicit verbal ToM task and on three levels of applied ToM abilities, early, basic, and advanced. These differences persisted even after controlling variables that have been shown to play an essential role in ToM development in children with ASD, such as the language level (Steele et al., 2003) or working memory (Kouklari et al., 2019).

Additional important information stemmed from comparing the two ASD groups and the TD group on ToM skills. This analysis helped to determine the specific types of deficits in children with ASD. Thus, applied ToM skills distinguished between the children with ASD and their typically developing peers. Statistically significant differences between the TD group and the two groups of children with ASD, both those in the “Lower” group and those with “Higher ToM abilities,” were found on all three ToMI subscales (Early, Basic, and Advanced). Therefore, parents perceived that the two groups with ASD had more difficulties than the TD group in understanding basic emotions, distinguishing between the physical and mental, making second-order inferences, or making complex social judgments. In addition, the group with ASD and “Lower ToM abilities” showed worse competence than the TD group on understanding first- and second-order false beliefs, double deception, and figurative language, all of which are assessed on the ToM verbal subtest. This group with ASD and “Lower ToM abilities” also presented difficulties on the contextual task, obtaining significantly worse results than the ASD group with “Higher ToM abilities.” By contrast, on the two measures that assess the explicit component of ToM, the verbal and contextual tasks on the NEPSY, the ASD group with “Higher ToM abilities” had similar performance to the TD group.

Clearly, two profiles of children with ASD without ID have been differentiated, namely “Higher” and “Lower” ToM abilities, based on explicit and applied ToM knowledge. However, the effect sizes (see Table 1) show the greater weight of the verbal task (understanding the other’s point of view) and the Basic ToMI subscale (understanding mental terms of feelings and actions) in differentiating the two groups. Furthermore, when comparing the two ASD groups and the TD group, the verbal task also discriminates the ASD group with worse ToM skills from the other two groups.

In sum, of the two Clusters of children with ASD, Cluster 2 (“Lower ToM abilities”) showed generalized explicit and applied ToM impairments, whereas the impairment of Cluster 1 (“Higher ToM abilities”) was more specific. In general, children in the latter group performed well on explicit ToM tasks where they had time to process the information and were given clear instructions and even options to select the correct answer (Barendse et al., 2018). Their failures focused on effectively applying the conceptual knowledge to real life interactions, which could be due, at least in part, to difficulties in developing appropriate strategies in an often unpredictable and changing context. Therefore, the initial hypothesis was fulfilled: the profiles identified in children with ASD differed in the level of development of different ToM skills and the application of the skills to daily life, which, even in the best performing profile, showed a weaker development than in the group with TD.

A second aim of the study was to examine whether the identified clusters could be differentiated by testing external variables such as symptom severity, social/adaptive behavior, and pragmatic abilities. As expected, the profile that had the greatest problems with ToM abilities showed greater ASD symptom severity and worse socio-adaptive and pragmatic skills. In fact, the group with “Lower ToM abilities” was characterized by more severe ASD symptoms and poorer pragmatic skills, in terms of inappropriate communicative beginnings and deficits in coherence and interpretation of language depending on the context, among other indicators. This group also showed significantly less mastery of daily living skills and poorer adaptive skills than the “Higher ToM abilities” profile, which showed less widespread impairment. Our results corroborate previous findings that have linked the prevalence of ToM in ASD to the degree of autistic symptoms (Lerner et al., 2011; Hoogenhout and Malcolm-Smith, 2017) or to pragmatic and social competence (Tager-Flusberg, 2001; Mazza et al., 2017; Baixauli et al., 2019).

On measures of Peer problems and Prosocial behavior, the means of both the Cluster with “Lower ToM abilities” and the Cluster with “Higher ToM abilities” are in the borderline/abnormal range. These impairments include behaviors such as inappropriate affect, social isolation, and failure to initiate interactions with peers, cooperate, share, make friends, express empathy, or provide emotional support. However, the two Clusters of children with ASD without ID were not significantly differentiated by the behaviors rated on these two scales. Thus, our results suggest that the difficulties of children with ASD-ID with prosocial behavior or relations with peers cannot be explained solely by differences in ToM ability. Previous studies concluded that, although performance on ToM tasks is associated with different subtypes of prosocial behavior (helping, cooperating, and comforting), the magnitude of the association is relatively weak (Imuta et al., 2016). Moreover, no ToM types have predicted parent reported social functioning of their children with ASD (Altschuler et al., 2018), and no simple or direct relationship has been found between behavioral indices of ToM ability and everyday social interactions, as in friendships described by children with high-functioning ASD (Calder et al., 2013).

### Limitations and Future Directions

This research has some limitations that should be considered, and so the findings should be interpreted with caution. On the one hand, the implicit component of the ToM was not evaluated, which would have allowed a more complete profile of the mind-reading skills of the participants to be outlined. We are aware that the best information collection strategy would have been to involve different sources by using a variety of assessment measures (multi-method assessment). However, parents of children with ASD are a reliable source of information about their children’s ToM because they have the opportunity to observe them during real world social interactions (Hutchins et al., 2011; Lerner et al., 2011). Even more, the ToM Inventory filled out by parents in our study has shown to provide a broad view the child’s theory of mind abilities, which can help to identify different profiles and potential targets within and across domains (early, basic, and advanced) (Greenslade and Coggins, 2016). Moreover, observational measures of pragmatic and social competence were not used either, as they were only assessed through parental estimates. Given the dependence and contextual variability characterizing these skills, it would have been desirable to have information from other informants (teachers, for example) in other significant settings in the child’s life.

Similarly, the small sample size and the predominance of males are two aspects that restrict the generalization of the results to the population of girls with ASD. It is possible that, in general, girls show a better profile. In fact, whereas social impairments and mentalizing language are linked in boys with ASD, this link seems to be weaker in girls (Boorse et al., 2019). Therefore, studies with larger samples that include girls with ASD are needed in order to find out how ToM deficits are manifested in this population. Moreover, ToM is a dynamic construct influenced by individual experiences, for what it should also be analyzed the specific role of contextual factors that have an impact on the developmental trajectories of explicit and applied ToM skills: maternal mind-mindedness (Laranjo et al., 2010), quality of relationships with siblings (Prime et al., 2016), or peer interactions (Slaughter et al., 2015). Longitudinal studies may also be an avenue for future research that can provide a more complete and dynamic understanding of the interaction between ToM and other indicators of the functioning of people with ASD, for example, in terms of predictors and social outcomes.

### Implications

The findings of the present study raise several clinical considerations regarding the diagnosis and assessment of autism spectrum disorders. First, this study confirms that children with ASD without ID vary in their development of ToM abilities. It is reinforced the idea that ToM is a multifaceted range of skills that are not always impaired to the same grade in children wit ASD. One group of children could show a more severe profile, characterized by deficits in cognitive understanding of other people’s mental states and in applied behavioral aspects of ToM skills, whereas the impairments in the other group could be related to their competence in applying ToM skills. In any case, even the subgroup with better ToM abilities, whose performance on explicit ToM is equal to that of TD, does not seem to successfully deal with social interactions in daily life. In these situations, it is necessary to respond spontaneously to a variety of events, which requires more resources than when performing tasks in contexts with greater stimulus control. Hence, it is important to complement the assessment using ToM performance tasks with procedures that evaluate how children cope with real-world social interactions and capture different levels, that is, early, basic, and advanced ToM. In conclusion, we think the data provided in this study are valuable because they emphasize the usefulness of incorporating applied and observational measures of ToM abilities into diagnostic processes in ASD clinical practice.

Second, this research provides information about the dynamic relationship of ToM with other important social functioning domains, as suggested by neuro-constructivist approaches (Bennett et al., 2013). The ASD group with “Higher ToM abilities” presents better adaptive skills related to daily life and socialization, such as money management and pragmatic skills, and less ASD symptomatology. However, both groups (Lower and Higher ToM) continue to show problems with peers and deficits in prosocial behavior, suggesting that deficits in social awareness are not the only explanation for social behavior problems. Other factors such as low social motivation or lack of opportunities for interaction or specific interference responses (i.e., reduction in social behaviors) may be involved. In this regard, a comprehensive assessment will help to clarify whether social problems are due to a lack of social cognition or social performance or both, in order to tailor interventions accordingly.

Together, the profiles identified suggest that ToM is appropriately conceptualized as a continuum of skills as well as an ASD severity indicator of individual differences in social outcomes. Therefore, information about ToM profiles has both clinical and practical importance in the evaluation and design of interventions that fit the profiles of difficulties and potential of people with ASD. On the one hand, it evidences the need to use batteries that include a wide range of measures and task demands in order to capture individual differences. The objective will be to identify the map of lower mind skills, as well as more advanced abilities, in children with ASD. On the other hand, closely related to the above, ToM profiles highlight the need to design specific treatment targets that fit an individual’s particular profile in a highly complex domain. Even though each child with ASD may have a different social functioning level, active participation in mentalization tasks related to understanding the mental states of others may improve his/her social awareness. Improvements in the conceptual understanding of ToM, however, are not sustained or generalized to real-life social settings and interactions (Fletcher-Watson et al., 2014). Consequently, as Bennett et al. (2013) highlighted, social cognition-based interventions should be developmentally sensitive and ecologically valid, incorporating naturalistic settings and engaging parents, teachers, and peers as facilitators (Kasari et al., 2010). Although ToM can impact social skills, social experiences themselves, especially support from peer relationships, can provide richer opportunities for everyday social interaction in school-aged children with ASD (Rodda and Estes, 2018).

## Data Availability Statement

The raw data supporting the conclusions of this article will be made available by the authors, without undue reservation, to any qualified researcher.

## Ethics Statement

The studies involving human participants were reviewed and approved by Ethics Committee of University of Valencia. Written informed consent to participate in this study was provided by the participants’ legal guardian/next of kin.

## Author Contributions

AM, CB, and BR designed the research. CB, RG, and BR performed the evaluations. CB analyzed the data. IB, BR, and CB contributed to the final writing of the manuscript. All authors revised the manuscript.

## Conflict of Interest

The authors declare that the research was conducted in the absence of any commercial or financial relationships that could be construed as a potential conflict of interest.
